# Inconsistent Media Mediation and Problematic Smartphone Use in Preschoolers: Maternal Conflict Resolution Styles as Moderators

**DOI:** 10.3390/children9060816

**Published:** 2022-05-31

**Authors:** Hwajin Yang, Wee Qin Ng, Yingjia Yang, Sujin Yang

**Affiliations:** 1 School of Social Sciences, Singapore Management University, Singapore 178903, Singapore; hjyang@smu.edu.sg (H.Y.); wq.ng.2019@phdps.smu.edu.sg (W.Q.N.); yingjiayang@smu.edu.sg (Y.Y.); 2 Department of Psychology, Ewha Womans University, Seoul 03760, Korea

**Keywords:** inconsistent media mediation, parent–child conflict tactics, child’s problematic smartphone use, psychological aggression, physical assault

## Abstract

Previous studies suggest that inconsistent parenting leads to undesired consequences, such as a child’s defiant reactance or parent–child conflicts. In light of this, we examined whether mothers’ inconsistent smartphone mediation strategies would influence their children’s problematic smartphone use during early childhood. Furthermore, given that harsh parenting often escalates a child’s behavioral problems, we focused on parent–child conflict resolution tactics as moderators. One hundred fifty-four mothers (ages 25–48 years; M = 35.58 years) of preschoolers (ages 42–77 months) reported their media mediation and parent–child conflict resolution tactics and their child’s problematic smartphone use. We found that the positive association between the mother’s inconsistent mediation and their child’s problematic smartphone use was more pronounced when mothers relied on negative parent–child resolution tactics—i.e., psychological aggression and physical assault. Our findings provide vital theoretical and empirical insights into mother–child relational characteristics for the child’s problematic smartphone use.

## 1. Introduction

The rapid development of smartphone applications for young children [1] and smartphones’ user-friendly interface [2] have granted children easy and convenient access to smartphones. The average amount of time spent on smartphones by young children aged 8 and under tripled from 15 min a day in 2013 to 48 min in 2017 [3]. Despite the benefits of smartphones, a burgeoning body of literature hints at problematic smartphone use in young children [4]. Characterized by excessive or uncontrollable use and addictive-like tendencies [5], problematic smartphone use can have detrimental effects on young children’s cognitive and social development, e.g., [6,7,8]. Given that approximately one in five children (3 to 6 years old) engages in a form of problematic smartphone use [4] and this rate increases every year [9], it is critical that we investigate the factors that may intensify or diminish children’s problematic smartphone use.

During early childhood, parents form the immediate environment in which a child develops [10]. Furthermore, given that children use smartphones within the context of family norms [11], parental factors such as parental media mediation—i.e., a set of strategies used to mitigate the potential negative effects of excessive media use [12]—likely play a substantial role in influencing children’s problematic smartphone use, e.g., [13]. Previous studies on this subject, however, have reported mixed findings. For instance, Collier et al. [14] suggest that parental mediation strategies may be effective in mitigating the excessive use of media (e.g., the internet, movies/videos, television, and video games) in children (0 to 12 years old) and adolescents (13 to 17 years old). Similarly, studies that examine parental smartphone mediation in relation to problematic smartphone use in children (10 years old and below) have shown that active and restrictive mediation served as a protective factor against problematic smartphone use [15,16]. In contrast, other studies have found that active and restrictive maternal mediation did not affect children’s (10 to 12 years old) problematic smartphone use [17], and that restrictive mediation may even increase its likelihood in children and adolescents aged 10 to 18 years old [18,19].

These mixed findings regarding media mediation can be attributed to several notable differences, such as a wide variety of media platforms (TV, internet, smartphone devices, etc.) that have been tested in previous studies. Furthermore, since most previous studies have focused on problematic smartphone use during middle/late childhood or adolescence, relatively less is known about early childhood despite the fact that young children appear to be more vulnerable to problematic smartphone use than older children due to cognitive immaturity and a lack of motivation for behavioral change and emotional self-regulation skills. More critically, there is a dearth of research on crucial parent–child relational factors that may account for the mixed findings by moderating the association between parental mediation and the child’s problematic smartphone use.

Given these results, our primary research goals were twofold. First, we sought to examine how the mother’s use of different mediation strategies—restrictive and inconsistent—would influence a child’s problematic smartphone use during early childhood. In particular, given that relatively scant attention has been paid to the inconsistent way in which parental mediation is communicated and implemented in terms of the child’s smartphone use [20], we focused on an inconsistent mediation strategy in which mediation rules are applied erratically. Furthermore, given that mothers typically spend more time with their children than fathers, usually take on a larger share of responsibility for caring for the child [21,22], and are more likely to use smartphones to “babysit” their children than fathers [23], we focused on maternal, instead of paternal, mediation strategies.

Second, to resolve mixed findings in the literature, we focused on parent–child conflict resolution styles as moderators. Two lines of theoretical and empirical evidence suggest close interrelations between parents’ inconsistent mediation, parent–child conflicts, and a child’s problem behaviors. Specifically, the first line of evidence sheds light on the link between maternal inconsistent mediation and a child’s problematic smartphone use. For instance, Gardner [24] suggests that inconsistent parental rules are relatively ineffective in regulating children’s behaviors and can lead to problematic behaviors. This is because when parents display inconsistent mediation for their child’s media use [25], children are less likely to internalize the rules and understand the consequences of their actions [26], and therefore respond with noncompliance and unwanted outcomes such as problematic smartphone use [27]. The second line of evidence elucidates the moderating role of mother–child conflict resolution strategies. Kildare and Middlemiss [28] showed that parents’ inconsistent mediation likely provokes anger, noncompliance, and active protest in young children, which, in turn, engenders mother–child conflicts. When the mother’s inconsistent mediation regarding the child’s smartphone use was coupled with negative mother–child conflict resolution tactics—such as physical assault or psychological aggression—the relationship between the mother’s inconsistent mediation and the child’s problematic smartphone use can be exacerbated to a greater extent [29]. Taken together, it is plausible that negative mother–child conflict resolution tactics may serve to moderate the link between the mother’s inconsistent mediation and the child’s problematic smartphone use.

### 1.1. Parental Mediation Strategies and the Child’s Smartphone Use

Broadly speaking, parents typically adopt a restrictive, active, or co-viewing mediation strategy [30,31]. Restrictive mediation involves the setting of rules concerning access to and/or time spent on media devices; active mediation involves parent–child discussions about media content; and co-viewing occurs when parents consume media with their children [30,31].

Multiple studies have investigated the relationship between parental mediation and problematic smartphone use in children, but their findings are mixed. Nielson et al. [32] conducted a systematic literature review on adolescents aged 12 to 19 years old and found that none of the three parental mediation strategies—active, restrictive, or co-viewing—were consistently associated with an increase or decrease in problematic internet use and problematic online gaming. Similarly, Lee et al. [17] found that parental mediation—specifically, that of mothers—had no significant effect on children’s (10 to 12 years old) problematic smartphone use. Recently, however, Yang et al. [33] studied children aged 10 to 12 years and found that the use of active mediation significantly predicted less problematic smartphone use in children. Although less is known about the link between maternal mediation strategies and problematic smartphone use in younger children, previous studies highlight the importance of parental mediation. For instance, Piotrowski [34] found that parental media mediation strategies (restrictive and active) are positively associated with the consumption of educational media content by children aged 3 to 8 years old.

In view of these mixed findings, a critical question arises regarding the impact of parents’ reliance on inconsistent mediation strategies—irregular and unpredictable rules regarding the duration and content of media use, including smartphones [27,35]. For instance, parents may be strict at times but give in to their children at other times [20]. Inconsistent media mediation seems more pertinent for younger children than for older children, since smartphone devices are often used as babysitting tools [36]. Consistently, previous studies suggest that most parents are likely to combine mediation strategies, especially for younger children’s (aged 0 to 3 years) digital play [37]. Although scant theoretical or empirical attention has been devoted to studying the link between inconsistent maternal media/smartphone mediation strategies and problematic smartphone use in young children, a theoretical framework based on inconsistent parenting supports the relationship between maternal inconsistent mediation strategies and problematic smartphone use [24].

According to Gardner [24], inconsistent parental rules are relatively ineffective in regulating children’s behaviors and can contribute to problematic behaviors such as temper tantrums and displays of defiance. Receiving erratic and inconsistent messages or consequences can confuse the child regarding which guidelines to follow [38]. Inconsistent parenting also makes it difficult for parents to impose intended consequences due to the unpredictability of rules—and, as a result, reinforces the child’s noncompliance [39]. Previous studies on inconsistent parenting suggest that parental inconsistency is detrimental for the child and leads to socioemotional and behavioral issues, e.g., [24,26]. Given that parents who are inconsistent in their parenting are likely to also be inconsistent in their mediation of media use [25], mothers’ reliance on inconsistent smartphone mediation strategies may lead to a reinforcement trap, whereby the short-term benefits (e.g., peace and conflict avoidance) of a parent’s yielding to a child’s request to use smartphones are gained at the cost of strengthening the child’s problematic behavior [24,40]. As a result, the child is less likely to internalize the rules and understand the consequences of their actions [26], which may increase unwanted outcomes such as problematic smartphone use [27]. Consistent with these notions, prior studies have found that inconsistent parenting is positively associated with problematic internet use in primary school children [25] and adolescents [41]; Martins et al. [26] found that the combination of inconsistent restrictive and active mediation significantly predicted greater social media use in adolescents.

Furthermore, previous studies on inconsistent parenting suggest that when parents sometimes respond harshly to the child and at other times permissively, such inconsistent responsiveness could cause the child to develop a sense of insecurity, i.e., insecure attachment [42], the downstream effects of which may extend to addictive behaviors [43]. Since individuals with an insecure attachment are more likely to experience inconsistent responsiveness, emotional instability, and distress, they may compensate for these inadequacies through excessive attachment to their smartphone [44]. In support of this proposition, Schmmenti et al. [45] have found that inconsistent parenting is associated with insecure attachment in adolescents, which was subsequently associated with internet use disorders. Applying this theoretical account to young children who experience inconsistent parenting, it is plausible that such children may rely more on smartphone devices as objects of attachment that provide immediate responsiveness and a sense of security [46].

Despite the empirical importance of inconsistent maternal smartphone mediation for young children’s problematic smartphone use, little is known about their relationship. Furthermore, most previous studies have focused on either preadolescent or adolescent children; few have considered children in their early childhood years—a critical period for the child’s future cognitive, linguistic, and socioemotional development [47]. Hence, we investigated the relationships between maternal smartphone mediation strategies and problematic smartphone use in preschoolers.

### 1.2. The Moderating Role of Parent–Child Conflict Resolution Tactics

To resolve mixed findings in the literature regarding the relationship between parental media mediation and children’s problematic smartphone use, we used mother–child conflict resolution styles as moderators. When dealing with conflicts, parents may employ either positive or negative conflict resolution tactics. Positive tactics include nonviolent discipline and reasoning, and negative tactics include psychological aggression and physical assault [48]. When parents use positive conflict resolution tactics, such as negotiation and compromise, children are less likely to exhibit internalizing and externalizing behaviors [49]. For instance, studies have shown that children aged 4 to 6 years whose parents have positive parent–child interactions or disciplinary parenting styles are less likely to develop internet addiction [50] and use media less at night [51], respectively. Given this, it is plausible that positive conflict resolution tactics would attenuate the relationship between inconsistent mediation and problematic smartphone use in children.

Conversely, when parents employed negative conflict resolution tactics, such as aggression and displays of anger, children were more likely to exhibit both internalizing and externalizing behaviors, e.g., [29,49,52]. In a similar vein, studies suggest that negative conflict resolution tactics have been related to a host of poor developmental outcomes in children [53,54,55,56,57], including greater levels of aggression, delinquency, interpersonal problems at school and home, and smartphone addiction [17,33]. Specifically, although intended to induce compliance with rules, psychological aggression, such as frequent negative commands and threats, may be ignored by the child, which results in passive noncompliance [58] or even acts of defiance [39,59]. Similarly, physical assault, such as pinching and hitting, has been linked to noncompliant behaviors [60,61]. Although physical assault is usually employed to enforce immediate compliance, it frequently leads to more negative outcomes, such as internet addiction [53].

Despite empirical support for the association of parent–child conflict resolution tactics and undesirable developmental outcomes in children, no study has empirically investigated the moderating role of parent–child conflict resolution tactics in the relationship between maternal mediation and children’s problematic smartphone use. Therefore, we aimed to fill this gap. In particular, we focused on mother–child conflict resolution strategies, since previous studies showed that mothers’ harsh parenting affected children’s emotional regulation more strongly than fathers’ [62]; maternal coercion was also associated with overt and relational aggression in preschoolers [63].

### 1.3. The Present Study

We focused on two smartphone-mediation strategies (restrictive and inconsistent), because they are regarded as the most prevalent mediation strategies. We omitted active mediation because it entails active discussion with the child regarding smartphone use, which may still be challenging for young children. We also omitted maternal co-viewing mediation, since there is a dearth of research on that strategy in relation to a child’s problematic smartphone use; thus, it would be difficult to formulate specific theoretical and empirical predictions. Concerning parent–child conflict resolution tactics, we focused on both positive (nonviolent discipline) and negative (psychological aggression and physical assault) tactics; we also removed reasoning, because it implicates in-depth verbal communication and reasoning, which are taxing for young children.

We hypothesized that maternal restrictive mediation would be associated with the child’s lower proneness to problematic smartphone use, whereas maternal inconsistent mediation would be associated with the child’s greater proneness to problematic smartphone use. For moderating relations, we hypothesized that negative mother–child conflict resolution tactics (i.e., psychological aggression and physical assault) would intensify the positive links between maternal inconsistent mediation strategies and the child’s problematic smartphone use. However, we expected that positive mother–child conflict resolution (nonviolent discipline) would attenuate the link between inconsistent maternal mediation and a child’s problematic smartphone use. Similarly, we hypothesized that positive (nonviolent discipline) conflict resolution tactics would reinforce the effectiveness of restrictive smartphone mediation for the child’s problematic smartphone use, whereas negative conflict resolution tactics (psychological aggression and physical assault) would decrease the effectiveness of restrictive mediation and aggravate the child’s problematic smartphone use.

## 2. Materials and Methods

### 2.1. Participants

For the study, 154 mothers (ages 25–48 years; M = 35.58 years, SD = 4.73) of preschoolers (ages 42–77 months; M = 61.42, SD = 8.93; 48.1% female) were recruited through an advertisement in local kindergartens. The majority of the mothers were Singaporean nationals (60.8%), Chinese in ethnicity (69.5%), married (92.9%), and college graduates (48.1%; see Table 1). Participants were compensated SGD 30 for their time.

### 2.2. Measures

**Media mediation**. We focused on three types of parents’ smartphone mediation strategies. Restrictive mediation (5 items; α = 0.606; e.g., “I have rules about my child’s time spent on smartphone devices”) was adapted from Nikken and Jansz’s [31] scale for the study’s context; the scale was developed to assess parental mediation of children’s time playing video games. Inconsistent mediation was measured using a single item adapted from the Perceived Parental Media Mediation scale [35] (“I would tell my child that he/she is not allowed to use a smartphone, but my child knows that the next time, he/she will be allowed to use it”). Mothers rated the frequency of both mediation strategies on a 5-point Likert scale (1 = *never*; 5 = *always*).

**Parent–child conflict resolution tactics**. We adapted a modified version of the Parent–child Conflict Tactics Scale (CTSPC) [48], which was designed to assess maternal resolution tactics when mothers experience conflict with or hostility toward their child; the scale has been widely used in the Asian context, e.g., [64,65,66]. The original scale consists of four subscales, but we only used three: (a) psychological aggression (5 items; α = 0.651; e.g., “I threatened to spank my child but did not actually do it”); (b) physical assault (4 items; α = 0.834; e.g., “I spank my child”); and (c) nonviolent discipline (4 items; α = 0.594; e.g., “I put my child in time out”). We did not use the reasoning subscale because that resolution tactic may not be age-appropriate, due to its reliance on extensive verbal discussion with the child. Mothers were instructed to report the frequency of relevant tactics for the past 6 months, using a 7-point Likert scale (0 = *never*; 1 = *once*; 2 = *twice*; 4 = *3–5 times*; 8 = *6–10 times*; 15 = *11–20 times*; 25 = *more than 20 times*).

**Problematic smartphone use**. To assess a tendency toward problematic smartphone use in children, we adapted Kwon et al.’s [67] smartphone addiction scale (short version), which was originally developed for adolescents. Of the 10 items from the original scale, we excluded five items that are not pertinent to young children (e.g., missing planned work due to smartphone use) and only retained items that describe typical smartphone-related problematic behaviors in young children (e.g., “My child feels impatient and fretful when she/he is not allowed to use their smartphone”). Mothers were instructed to rate their child’s behaviors on a 6-point Likert scale (1 = *strongly disagree*; 6 = *strongly agree*). The adapted scale had good reliability (α = 0.907). Summed scores were computed, with higher scores reflecting greater proneness to problematic smartphone use.

**Demographics**. Demographic information was collected using a background questionnaire. As covariates in our hypothesized moderation model, we used the child’s age (in months) and sex; mother’s age (in years); education (as a proxy for socioeconomic status) [68,69]; ethnicity (Chinese, Malay, Indian, Caucasian, and Other); and, as a proxy for family type, marital status (1 = *married*, 2 = *divorced*, 3 = *separated*, 4 = *other*). Mother’s education was rated based on a scale from 1 (*Primary school*) to 8 (*Doctoral degree*; e.g., Ph.D. or J.D.). These covariates were included in line with previous studies that suggest they are significantly associated with smartphone overuse. For instance, studies suggest that younger children, e.g., [70] and females [71] are at higher risk for problematic smartphone use, and parents’ education is positively associated with smartphone addiction [67]. Another line of research suggests that females tend to exhibit greater smartphone addiction than males [72].

### 2.3. Procedure

Mothers completed a questionnaire online or on paper regarding their media mediation attitude and mother–child conflict resolution tactics and their child’s problematic smartphone use. The study’s procedure was approved by the institutional review board of Singapore Management University (IRB approval number IRB-16-060-A089(916) 25 September 2018).

## 3. Results

Descriptive statistics and bivariate zero-order correlations are shown in Table 1. We found no evidence of multicollinearity among variables; all VIF values (<2.48) were within an acceptable range. To examine the relationship between a mother’s mediation strategies and a child’s proneness to problematic smartphone use, we performed an ordinary least squares (OLS) regression analysis. We regressed a child’s problematic smartphone use on covariates (the child’s age and sex and mother’s age, ethnicity, marital status, and education); two mediation strategies (restrictive and inconsistent); and the three parent–child conflict tactics (nonviolent discipline, psychological aggression, and physical assault). Overall, the model was significant and explained a significant amount of variance in a child’s problematic smartphone use, *R*^2^ = 0.242, *F* = 2.877, *p* < 0.001 (see Table 2). For predictors, we found that inconsistent mediation positively predicted a child’s problematic smartphone use (B = 1.570, SE = 0.408, *p* < 0.001), while restrictive mediation negatively predicted a child’s problematic smartphone use, B = −2.178, SE = 0.730, *p* = 0.003. Of the three parent–child conflict tactics, only physical assault significantly predicted a child’s problematic smartphone use, B = −0.109, SE = 0.047, *p* = 0.020 (see Table 2). For covariates, only maternal education was significant, B = −0.558, SE = 0.273, *p* = 0.043.

Next, we conducted a series of moderation analyses using the PROCESS macro (Model 1) for SPSS [73]. We examined whether a mother’s parent–child conflict resolution tactics (nonviolent discipline, psychological aggression, and physical assault) would moderate the relationship between the mother’s smartphone mediation strategies (restrictive and inconsistent) and their child’s proneness to problematic smartphone use. The child’s age and sex and the mother’s age, education, ethnicity, and marital status were included as covariates.

**Figure 1:**
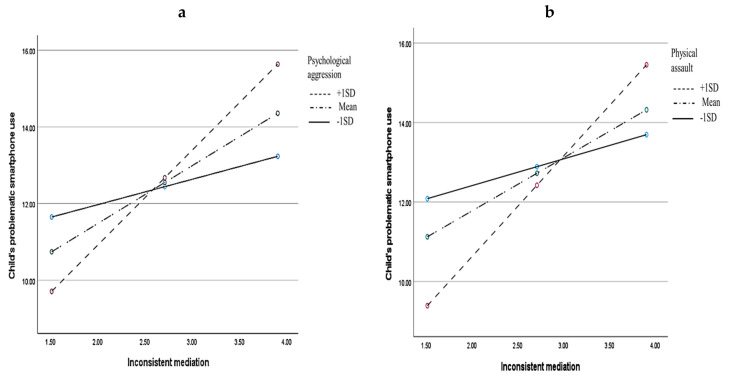
Simple slopes analysis illustrating the moderating effects of parent–child conflict resolution tactics—psychological aggression (**a**) and physical assault (**b**)—on the relationship between inconsistent maternal mediation strategies (psychological aggression and physical assault) and the child’s problematic smartphone use.

We found that the mother’s inconsistent media mediation significantly interacted with physical assault, *F* (1, 143) = 12.947, *p* = 0.0004, in explaining the child’s problematic smartphone use. Similarly, the mother’s inconsistent media mediation significantly interacted with psychological aggression in predicting a child’s problematic smartphone use, *F* (1, 143) = 7.729, *p* = 0.006 (see Table 3). Further scrutiny using simple slopes analysis confirmed that the positive relationships between inconsistent mediation and the child’s problematic smartphone use were more pronounced at higher levels of both negative conflict resolution tactics—psychological aggression (see Figure 1a) and physical assault (see Figure 1b). In contrast, nonviolent discipline as a conflict resolution tactic did not interact with the mother’s inconsistent mediation strategy (*p* = 0.342). Moreover, none of the parent–child conflict resolution tactics interacted with a restrictive mediation strategy in explaining a child’s problematic smartphone use, all *p*s > 0.240.

## 4. Discussion

Our primary finding is that negative mother–child conflict resolution tactics—i.e., psychological aggression and physical assault—moderated the relationship between inconsistent maternal media mediation and young children’s problematic smartphone use during early childhood. However, restrictive mediation interacted with neither positive nor negative mother–child conflict resolution tactics in explaining children’s problematic smartphone use, although it significantly predicted lower proneness to problematic smartphone use by children. Consistent with theoretical accounts of inconsistent parenting [24] and insecure attachment [74,75], our results highlight an adverse developmental outcome (i.e., excessive and addictive smartphone use in children) that is closely associated with mothers’ inconsistent mediation strategies and negative conflict resolution methods during early childhood.

Several findings merit further discussion. Our results whereby different maternal mediation strategies play vital roles in a child’s problematic smartphone use are consistent with previous studies that emphasize the importance of parental mediation in influencing the child’s media/smartphone use [14,16,62]. In particular, it is notable that restrictive and inconsistent maternal mediation strategies influence a child’s smartphone use in different directions; the former attenuates and the latter aggravates a child’s problematic smartphone use. Our results imply that inconsistent mediation strategies for children’s smartphone use interfere with children’s internalizing rules and understanding the consequences of their actions [26], and thereby predict higher proneness to problematic smartphone use.

Previous studies on parental mediation strategies for children’s smartphone use have been limited, because the majority have focused on either active or restrictive mediation strategies and either preadolescents or adolescents [16,62]; these studies have yielded mixed findings and little knowledge about young children’s smartphone use. To our knowledge, our study is the first to emphasize the danger of an inconsistent maternal mediation strategy and its interaction with negative parent–child conflict resolution tactics in worsening preschoolers’ problematic smartphone use. Given our finding that maternal inconsistencies in exercising a specific mediation strategy are detrimental for a child’s problematic smartphone use, previous studies’ mixed findings regarding the effectiveness of either active or restrictive strategies for a child’s smartphone use may be the result of a lack of focus on the extent of consistency in applying mediation strategies.

Furthermore, given that we did not find that positive conflict resolution tactics (i.e., nonviolent discipline) interacted with restrictive mediation strategies in explaining the child’s problematic smartphone use, our results suggest that negative conflict resolution tactics appear to be more impactful for young children than positive conflict resolution tactics in giving rise to the child’s problematic smartphone use. This corroborates previous studies that highlight more pronounced negative developmental outcomes associated with negative resolution tactics, e.g., [33,53,56].

Given that the primary goal of our research was to examine the moderating role of mother–child conflict resolution strategies, our findings should not be interpreted as identifying the psychological mechanism (i.e., process) that underlies the relationship between maternal smartphone mediation and a child’s problematic smartphone use. However, in view of the previously established link between inconsistent parenting, insecure attachment, and addictive behaviors in children [43,74,75], future studies should examine whether a child’s insecure attachment, which is bolstered by inconsistent parenting, exacerbates their dependence on smartphones to compensate for feelings of inadequacy (e.g., emotional instability or distress) through excessive attachment to their smartphone [44].

Our study is not without limitations. First, given that we relied on a single-item measure of an inconsistent maternal smartphone mediation strategy, future studies should replicate our findings by using a more age-appropriate and well-established scale, which is not yet available in the literature. Although single-item scales are useful for representing a global construct, they are limited in terms of shedding light on different facets of parents’ inconsistent smartphone mediation strategies. Thus, a more fine-grained and age-appropriate measure of inconsistent smartphone mediation is warranted.

Second, although not necessarily a major limitation, given that our sample only included mothers of preschool children, it is difficult to generalize our findings to fathers, whose media/smartphone mediation and conflict resolution strategies may have different developmental implications for the child’s problematic smartphone use. Furthermore, given that both fathers and mothers are the primary sources of social interaction during early childhood, future studies are warranted to examine how consistency/inconsistency between the two parents’ media mediation and conflict resolution strategies would influence the child’s problematic smartphone use.

In a similar vein, caution is advised when generalizing our findings to children of different ages, especially preadolescents or adolescents for whom parental media mediation or parent–child conflict resolution strategies have different developmental implications. Given that parents’ mediation and conflict resolution tactics may have different repercussions for older children, it is plausible that the interaction effects of maternal mediation and negative parent–child conflict resolution strategies may manifest themselves to somewhat different extents. For instance, given that adolescents tend to prioritize autonomy from parents, parents’ restrictive mediation may not be effective; instead, it may backfire and exacerbate the child’s problematic smartphone use, especially when they resort to negative parent–child conflict resolution tactics. Future studies are therefore needed to shed light on how age would modulate the relationship between parental media mediation and negative conflict resolution tactics in elucidating problematic smartphone use in children.

Our findings have several important practical implications for parents. First, our study emphasizes that parents play an important role in guiding and regulating their children’s smartphone use. However, not all mediation strategies are equally effective in protecting against problematic smartphone use in children. Parents may be encouraged to use restrictive smartphone mediation, given that it seems to be effective for preventing problematic smartphone use in young children. Because children have immature self-control, restrictive mediation is particularly suitable for controlling young preschoolers’ impulses through external rules [76]. On the other hand, parents should refrain from using inconsistent mediation, in which rules are applied erratically, to regulate a child’s smartphone use.

Second, it is crucial that parents understand that several facets of their parenting (e.g., media mediation) and parent–child relational characteristics can combine and lead to unfavorable developmental outcomes for a child’s problematic smartphone use. Parents’ inconsistent mediation strategies, coupled with the use of negative conflict resolution styles to more easily compel the child’s compliance, could actually engender unexpected and unwanted results—i.e., problematic smartphone use in children. Hence, more holistic interventions for families and parents are needed to help protect children from falling prey to potential risks associated with digital media use. In a related vein, given the detrimental impacts of both inconsistent smartphone mediation and negative mother–child conflict resolution tactics, it is important to identify factors that shape these parental characteristics in order to design a more holistic family-oriented intervention for children at risk. In particular, contextual factors that intensify parental stress—which, in turn, influences their smartphone-mediation and conflict-resolution strategies—are worthy of further attention. For instance, the outbreak of COVID-19 pandemic has affected many facets of family life in a complex manner (e.g., parents’ working from home with children) and triggered changes in family relationships. Although we do not have any data to test this subject, future studies should examine how contextual factors, such as stressful pandemic situations or the mother’s employment status (e.g., night shift) exacerbate maternal smartphone mediation and conflict resolution styles.

In sum, despite the increasing use of smartphone devices by young children, little was previously known about the impacts of parental (especially maternal) mediation strategies (inconsistent or restrictive) on children’s problematic smartphone use during early childhood. Given this, our findings underscore the critical need for parents to implement effective and consistent smartphone mediation strategies as early as possible to protect young children against unhealthy overreliance on smartphone devices. Moreover, less attention has been paid to factors that may modulate the above relation. Our research identifies the hazardous interactive effects of inconsistent maternal smartphone mediation and negative mother–child conflict resolution tactics (such as psychological aggression or physical assault) in escalating problematic smartphone use in young children. The outcomes of this research are valuable for developing family-centered intervention strategies to protect children from overreliance on smartphone use from early childhood, especially in view of the negative cognitive and socioemotional outcomes of smartphone overuse [6,7]. Furthermore, these findings provide useful guidelines for parents, caregivers, and educators regarding the need for appropriate regulation of media consumption during early childhood.

## Figures and Tables

**Table 1:** Descriptive Statistics and Bivariate Zero-order Correlations.

	*M*	*SD*	Range	1	2	3	4	5	6	7	8	9
1. Child’s age (months)	61.42	8.93	42–77	-								
2. Mother’s ethnicity (% Chinese) ^1^	69.5		1–5	-								
3. Mother’s marital status (% married) ^2^	92.2		1–4	-								
4. Child’s sex (% boys) ^3^	51.90		1–2	−0.029	-							
5. Mother’s age (years)	35.58	4.73	25–48	−0.022	−0.043	-						
6. Mother’s education ^4^	4.96	1.88	1–9	−0.027	−0.018	0.188 *	-					
8. Restrictive mediation	3.40	4.28	1.6–5	−0.129	−0.151	0.150	0.171 *	-				
9. Inconsistent mediation	2.69	1.26	1–5	−0.029	0.020	−0.036	−0.140	0.043	-			
10. Psychological aggression	15.31	17.71	0–125	0.021	0.005	−0.170 *	−0.025	0.042	0.104	-		
11. Physical assault	8.24	15.06	0–100	−0.021	0.069	−0.281 **	−0.028	−0.094	−0.035	0.699 **	-	
12. Nonviolent discipline	28.31	19.59	0–90	−0.087	−0.148	0.036	0.115	0.312 **	−0.025	0.526 **	0.417 **	-
13. Child’s problematic smartphone use	12.67	6.18	5–30	0.034	0.014	−0.076	−0.235 **	−0.198*	0.341 **	0.063	−0.068	−0.018

*Note*. ^1^ Ethnicity was a nominal variable coded 1 for *Chinese (69.5%)*; 2 for *Malay (3.2%)*; 3 for *Indian (15.6%)*; 4 for *Caucasian (0.6%)*; and 5 for *Other (10.4%)*. ^2^ Marital status was a nominal variable coded 1 for *married (92.2%)*; 2 for *divorced (3.9%)*; 3 for *separated (1.9%)*; and 4 for *Other (1.3%)*. ^3^ Sex was coded 1 for boys and 2 for girls. ^4^ Education was reported on a scale ranging from 1 = *Primary school to* 8 = *Doctoral or other professional degree*. * *p* < 0.05; ** *p* < 0.01.

**Table 2:** Ordinary Least Squares Regression for a Child’s Problematic Smartphone Use.

	B	SE B	*β*
Child’s age (months)	0.018	0.055	0.025
Child’s sex ^1^	0.303	0.977	0.025
Mother’s age	−0.058	0.106	−0.045
Mother’s education	−0.558	0.273	−0.167 *
Ethnicity ^2^			
Chinese	1.455	1.501	0.109
Malay	2.639	2.970	0.077
Indian	1.085	1.852	0.063
Marital status ^3^			
Married	2.236	2.976	0.095
Divorced	−1.697	3.867	−0.054
Separated	3.774	6.441	0.050
Restrictive mediation	−2.178	0.730	−0.250 **
Inconsistent mediation	1.570	0.408	0.305 **
Psychological aggression	0.058	0.042	0.166
Physical assault	−0.109	0.047	−0.269 *
Nonviolent discipline	0.032	0.032	0.102

*Note*. *R*^2^ = 0.218. Adjusted *R*^2^ = 0.168. * *p* < 0.05; ** *p* < 0.01. ^1^ Sex was coded 1 = boys and 2 = girls. ^2^ Ethnicity was dummy coded with reference to the last category (“Other”). ^3^ Marital status was dummy coded with reference to the last category (‘Other”).

**Table 3:** Conditional Indirect Effects of Maternal Mediation on Child’s Problematic Smartphone Use.

	Coefficients	*SE*	*t*	*p*	Confidence Interval
Inconsistent Mediation					
Inconsistent mediation (IM)	0.769	0.474	1.622	0.107	[−0.169, 1.706]
Physical assault	−0.239	0.658	−3.629	0.0004	[−0.369, −0.109]
IM × Physical assault	0.081	0.023	3.598	0.0004	[0.035, 0.126]
Inconsistent mediation (IM)	0.610	0.568	1.074	0.285	[−0.513, 1.733]
Psychological aggression	−0.170	0.070	−2.422	0.017	[−0.308, −0.031]
IM × Psychological aggression	0.067	0.024	2.780	0.006	[0.019, 0.115]
Inconsistent mediation (IM)	1.152	0.727	1.584	0.116	[−0.286, 2.589]
Nonviolent	−0.049	0.061	−0.796	0.428	[−0.170, 0.072]
IM × Nonviolent	0.020	0.021	0.954	0.342	[−0.022, 0.062]
Restrictive Mediation					
Restrictive mediation (RM)	−2.202	0.872	−2.523	0.013	[−3.927, −0.478]
Physical assault	−0.191	0.129	−1.481	0.141	[−0.446, 0.064]
RM × Physical assault	0.044	0.038	1.159	0.248	[−0.031, 0.119]
Restrictive mediation (RM)	−1.795	0.959	−1.871	0.064	[−3.691, 0.102
Psychological aggression	−0.013	0.120	−0.112	0.911	[−0.250, 0.223]
RM × Psychological aggression	0.009	0.032	0.289	0.773	[−0.055, 0.073]
Restrictive mediation (RM)	−1.670	1.178	−1.418	0.135	[−3.997, 0.659]
Nonviolent	0.025	0.124	0.198	0.844	[−0.222, 0.271]
RM × Nonviolent	−0.002	0.033	−0.046	0.964	[−0.066, 0.063]

## Data Availability

Data will be available upon request.
